# The Effectiveness of Pleural Cholesterol Levels in Differentiating Transudate from Exudate and Comparison with Light’s Criteria

**DOI:** 10.3390/life16071195

**Published:** 2026-07-19

**Authors:** Aziz Gumus, Elvan Senturk Topaloglu, Neslihan Ozcelik, Abdurrahman Kotan, Hasan Veysel Keskin, Omer Topaloglu, Songul Ozyurt

**Affiliations:** 1 Department of Pulmonology, Faculty of Medicine, Recep Tayyip Erdogan University, 53100 Rize, Turkey; aziz.gumus@erdogan.edu.tr (A.G.); neslihan.ozcelik@erdogan.edu.tr (N.O.); songul.ozyurt@erdogan.edu.tr (S.O.); 2 Department of Pulmonology, Erzurum Regional Training and Research Hospital, 25050 Erzurum, Turkey; kotanmd128@gmail.com; 3 Department of Pulmonology, Ahi Evren Thoracic and Cardiovascular Surgery Training and Research Hospital, Trabzon University, 61040 Trabzon, Turkey; keskinh.veysel@gmail.com; 4 Department of Thoracic Surgery, Faculty of Medicine, Recep Tayyip Erdogan University, 53100 Rize, Turkey; omer.topaloglu@erdogan.edu.tr

**Keywords:** pleural effusion, pleural cholesterol, exudate, transudate, Light’s criteria, thoracentesis

## Abstract

Background: Differentiating exudative from transudative pleural effusions is essential for accurate diagnosis and management. Although Light’s criteria remain the standard diagnostic method, they may incorrectly classify a substantial proportion of transudative effusions as exudates. This study aimed to evaluate the diagnostic performance of pleural fluid cholesterol and compare it with Light’s criteria. Methods: This retrospective study included 751 patients with pleural effusion who underwent diagnostic evaluation between 2015 and 2024 and had a definitive etiological diagnosis. All patients underwent ultrasound-guided thoracentesis with simultaneous pleural fluid and serum biochemical analyses. Based on final etiological diagnosis, effusions were classified as transudative or exudative. Results: The mean age was 68.6 ± 15.8 years. Of the pleural effusions, 516 (68.7%) were exudative and 235 (31.3%) were transudative. Malignant pleural effusion was the most common cause of exudates, whereas congestive heart failure predominated among transudates. Pleural fluid cholesterol levels were significantly higher in exudative than transudative effusions (91.1 ± 30.7 vs. 28.9 ± 13.5 mg/dL, *p* < 0.001). Receiver operating characteristic (ROC) analysis demonstrated excellent diagnostic performance for pleural fluid cholesterol (AUC = 0.976, 95% CI 0.965–0.987, *p* < 0.001). A pleural cholesterol cut-off value of ≥50 mg/dL demonstrated 92.2% sensitivity, 94.9% specificity, 97.5% positive predictive value, and 84.8% negative predictive value for identifying exudative effusions. Light’s criteria showed higher sensitivity (99.2%) but lower specificity (84.4%). Light’s criteria misclassified 15.6% of transudative effusions as exudates, whereas pleural cholesterol misclassified only 5.1%. Conclusions: Pleural fluid cholesterol measurement demonstrated high diagnostic accuracy in differentiating exudative and transudative pleural effusions and substantially reduced false exudate classification compared with Light’s criteria. Routine assessment of pleural cholesterol may improve the diagnostic evaluation of pleural effusions.

## 1. Introduction

Pleural effusion (PE) is a common clinical condition. Although there are more than 60 etiologic causes of PE, approximately 75% of the cases occur due to congestive heart failure (CHF), cancer, pneumonia, and tuberculosis [1,2]. It is important to make this distinction because the underlying mechanism, as well as subsequent management and treatment, depends on whether the effusion is exudative or transudative. The mechanism underlying transudative pleural effusion involves an increase in hydrostatic pressure or a decrease in plasma oncotic pressure. In contrast, the mechanism underlying exudative pleural effusion involves inflammation or impaired lymphatic drainage. Etiologic diagnosis can often be established without much difficulty. However, in 10–25% of cases, the cause of the effusion cannot be determined definitively despite the use of extensive diagnostic methods [3]. Sometimes, patients may have more than one etiologic cause of PE, such as the coexistence of heart failure and malignancy [4]. The first step in the etiologic diagnosis of any PE is to distinguish transudate from exudate [5]. As an initial step, fluid samples are obtained by thoracentesis performed with bedside ultrasonography, with low complication rates [6]. The use of Light’s criteria to differentiate exudative from transudative effusions was first described in 1972 and has continued to be used as the standard method for 54 years [7]. Although these criteria identify nearly all exudates accurately, they have the disadvantage of misclassifying approximately 25% of transudates as exudative fluid, particularly in patients with underlying congestive heart failure who are receiving diuretic therapy.

It has been suggested that pleural fluid cholesterol may be as effective as the components of Light’s criteria in distinguishing exudates from transudates [8]. An alternative diagnostic approach, known as the Heffner criteria, incorporates pleural fluid cholesterol, pleural fluid protein, and pleural fluid lactate dehydrogenase for the classification of pleural effusions [9]. Furthermore, pleural fluid cholesterol, either alone or in combination with other biochemical markers, has been reported to reduce the misclassification of pleural effusions compared with Light’s criteria [9]. It is still unclear why pleural fluid cholesterol is elevated in exudative fluids. It might rise in these fluids either due to the degeneration of leukocytes and erythrocytes in the pleural space or because it passes from pleural capillaries into the pleural cavity as a result of increased pleural permeability [10].

This study aimed to determine the effectiveness of pleural cholesterol in differentiating transudates from exudates and, in particular, to investigate its contribution to correct classification in patients who are etiologically transudative but are incorrectly identified as exudative.

## 2. Materials and Methods

### 2.1. Study Design and Patient Selection

**Figure 1:**
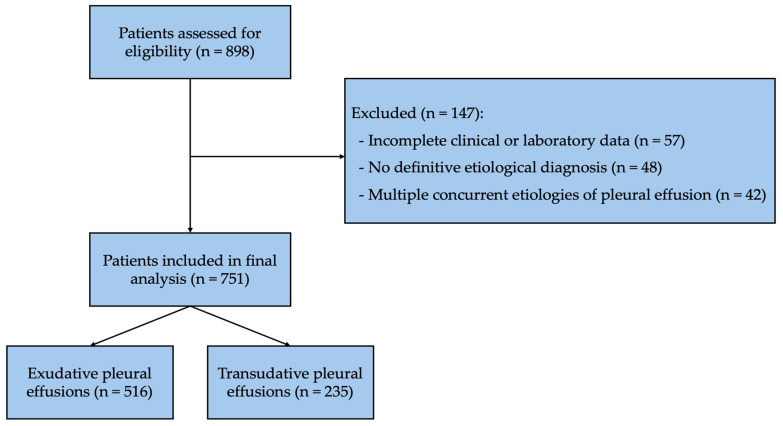
STARD flow chart illustrating patient selection and classification of pleural effusions included in the final analysis.

This retrospective single-center study included all consecutive adult patients who underwent ultrasound-guided diagnostic thoracentesis for pleural effusion at the Department of Pulmonology, Recep Tayyip Erdoğan University Training and Research Hospital between January 2015 and December 2024. A total of 898 patients were initially screened for eligibility. Fifty-seven patients with missing clinical or laboratory data, 48 patients without a definitive etiological diagnosis, and 42 patients with multiple concomitant etiologies for pleural effusion (e.g., congestive heart failure with pneumonia, congestive heart failure with malignancy, or malignancy with atelectasis) were excluded according to the predefined exclusion criteria. Consequently, 751 patients with a definitive etiological diagnosis were included in the final analysis. The patient selection process is summarized in Figure 1.

Fifty-seven patients with missing data, 48 patients in whom a definitive diagnosis could not be established, and 42 cases with more than one etiology (such as CHF–pneumonia, CHF–malignancy, and malignancy–atelectasis) were excluded from the study. The study was continued with 751 patients who had a definitive etiologic diagnosis.

Inclusion criteria:

- Patients older than 18 years
- Patients with a definitively established etiologic diagnosis of pleural effusion

Exclusion criteria:

- Patients under 18 years of age
- Patients with missing data
- Patients in whom a definitive clinical and etiologic diagnosis could not be established
- Patients diagnosed with hyperlipidemia and/or using antihyperlipidemic medications
- Patients diagnosed with chylothorax, pseudochylothorax, or hemothorax
- Patients with more than one disease that could lead to the release of both transudative and exudative fluid (such as the coexistence of cancer and atelectasis, cancer and hypoalbuminemia, or heart failure and pneumonia)

All patients with pleural effusion underwent diagnostic thoracentesis under ultrasonographic guidance. Cell count, lactate, protein, glucose, lactate dehydrogenase (LDH), cholesterol, and triglyceride measurements were performed in pleural fluid samples. Simultaneously, blood samples were obtained, and biochemical parameters, including protein and LDH, were analyzed. In all patients, the underlying primary disease-causing pleural effusion was investigated.

The final etiological diagnosis, which served as the reference standard for all diagnostic accuracy analyses, was established by the attending pulmonologist using all available clinical, radiological, microbiological, cytological, histopathological, and laboratory findings. Patients were classified into transudative or exudative pleural effusions according to the definitive underlying etiology rather than biochemical criteria alone. The etiological diagnosis of pleural effusion was confirmed according to the following predefined diagnostic criteria:

1. **Congestive heart failure:** Clinical and/or echocardiographic evidence of cardiac dysfunction, with or without cardiomegaly, consistent with congestive heart failure.
2. **Malignant pleural effusion:** Detection of malignant cells in pleural fluid cytology and/or histopathological confirmation of pleural malignancy.
3. **Parapneumonic effusion:** Clinically and radiologically confirmed pneumonia together with evidence of infection, including positive microbiological culture, positive sputum Gram stain, leukocytosis, or elevated C-reactive protein levels.
4. **Tuberculous pleural effusion:** Demonstration of *Mycobacterium tuberculosis* by acid-fast bacilli smear or culture from pleural fluid, or identification of necrotizing granulomatous inflammation on pleural biopsy after exclusion of other granulomatous diseases. A favorable clinical response to anti-tuberculosis treatment was also considered supportive evidence.
5. **Renal failure:** Chronic kidney disease with elevated serum urea (>20 mmol/L) or serum creatinine (>167 μmol/L), with or without clinical evidence of volume overload.
6. **Hypoalbuminemia:** Serum albumin level < 20 g/L.

At the same time, patients with pleural effusion were classified as transudate or exudate according to Light’s criteria. If one or more of the following conditions were present, the effusion was classified as exudative [5]:

- The ratio of pleural protein to serum protein (protein ratio) ≥ 0.5
- The ratio of pleural LDH to serum LDH (LDH ratio) ≥ 0.6
- Pleural LDH > two-thirds of the upper limit of normal serum LDH

### 2.2. Statistical Analysis

Statistical analyses were performed using IBM SPSS Statistics for Windows, version 27.0 (IBM Corp., Armonk, NY, USA). The conformity of continuous variables to normal distribution was assessed using the Kolmogorov–Smirnov test. Continuous variables were presented as mean ± standard deviation and median (interquartile range), while categorical variables were presented as number (%). For comparisons between two groups, Student’s t-test was used for variables with a normal distribution, and the Mann–Whitney U test was used for variables without a normal distribution. Categorical variables were compared using the chi-square test. A *p*-value of <0.05 was considered statistically significant in all analyses.

For each diagnostic parameter, sensitivity, specificity, positive predictive value (PPV), and negative predictive value (NPV) were calculated. Receiver operating characteristic (ROC) curve analysis was performed to evaluate diagnostic performance. The optimal pleural fluid cholesterol cut-off value was determined using the maximum Youden Index (sensitivity + specificity − 1), which identifies the threshold providing the best balance between sensitivity and specificity. The area under the ROC curve (AUC) with 95% confidence intervals was calculated for all evaluated parameters.

## 3. Results

The mean age of the cases was 68.6 ± 15.8 years. Of them, 291 (38.7%) were female and 460 (61.3%) were male. According to the etiologic classification, 516 (68.7%) pleural effusions were exudative and 235 (31.3%) were transudative. Among the etiologies causing exudates, malignant pleural effusion (MPE) ranked first with 314 cases (60.9%). Parapneumonic effusion was the second most common cause with 114 cases (22.1%), and tuberculosis-related effusion ranked third with 49 cases (9.5%). Among the etiologic causes of transudates, CHF ranked first with 201 cases (85.5%), followed by hypoalbuminemia in 14 cases (6.0%) and chronic renal failure in 11 cases (4.9%).

The clinical and laboratory characteristics of patients with transudative and exudative pleural effusions were compared in Table 1. Patients with transudative effusions were found to be significantly older. However, this group contained comparable numbers of males and females. All laboratory values, except serum lactate dehydrogenase, were significantly higher in exudative fluids than in transudative fluids.

**Figure 2:**
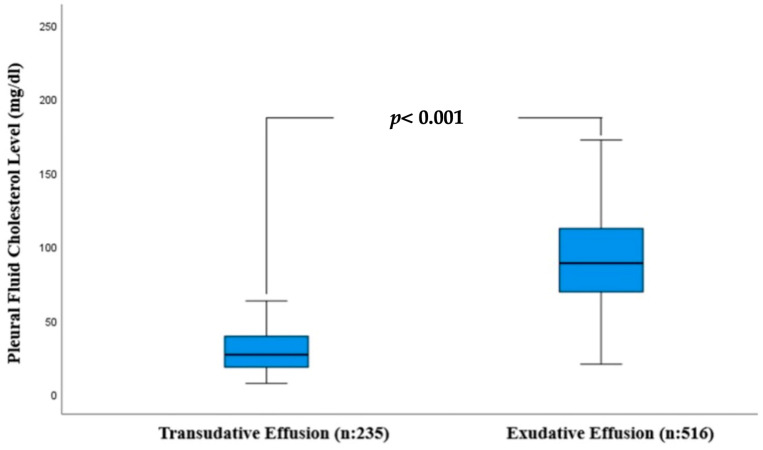
Box-and-whisker plot showing pleural fluid cholesterol levels in exudative and transudative pleural effusions. Pleural fluid cholesterol levels were significantly higher in exudative than in transudative pleural effusions (*p* < 0.001).

Pleural cholesterol levels were markedly higher in exudative fluid (91.1 ± 30.7 mg/dL) than in transudative fluid (28.9 ± 13.5 mg/dL) (Figure 2).

Pleural cholesterol levels were also evaluated according to the major etiological subgroups. The mean pleural cholesterol levels were 28.6 ± 13.6 mg/dL in congestive heart failure, 90.8 ± 29.7 mg/dL in malignant pleural effusion, 88.7 ± 32.6 mg/dL in parapneumonic effusion, and 95.6 ± 21.6 mg/dL in tuberculous pleural effusion. No significant differences were observed among the major exudative etiologies (malignant pleural effusion, parapneumonic effusion, and tuberculous pleural effusion), whereas pleural cholesterol levels were consistently lower in congestive heart failure, the predominant cause of transudative pleural effusions.

**Figure 3:**
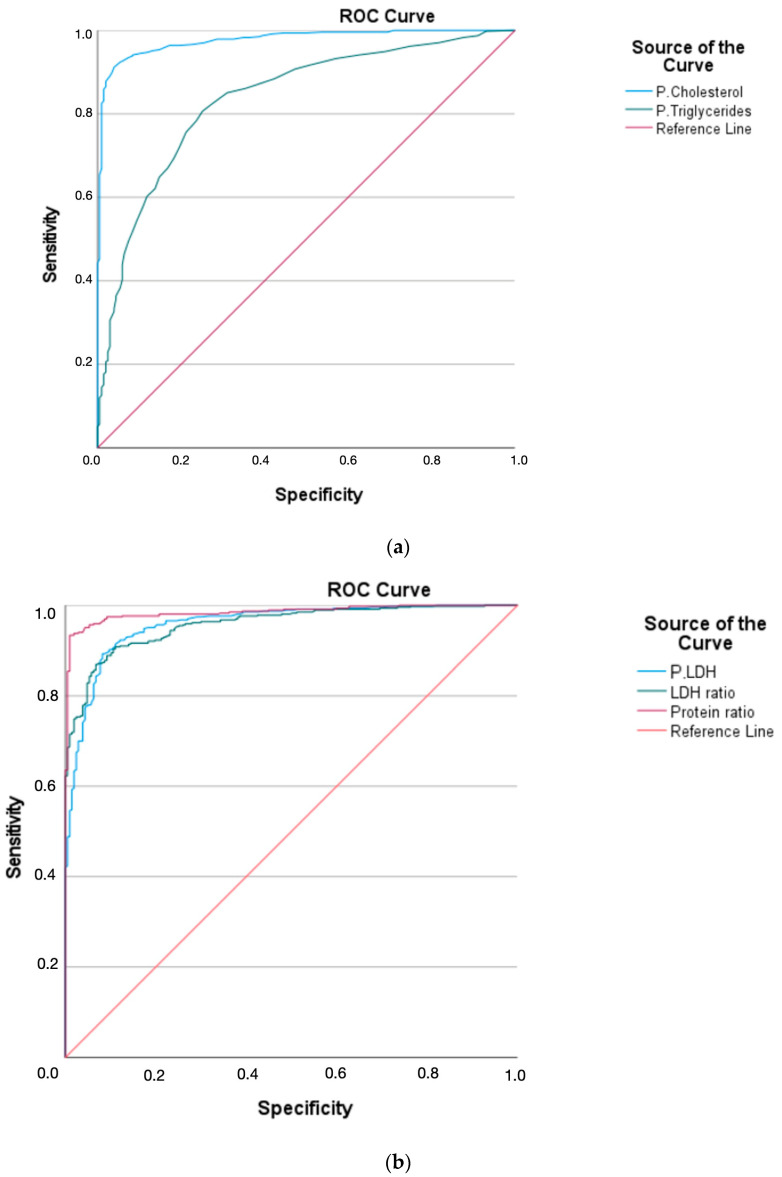
(**a**). Receiver operating characteristic (ROC) curves demonstrating the diagnostic performance of pleural fluid cholesterol and pleural fluid triglyceride levels for differentiating exudative from transudative pleural effusions. (**b**). Receiver operating characteristic (ROC) curves demonstrating the diagnostic performance of the components of Light’s criteria for differentiating exudative from transudative pleural effusions.

Using ROC curve analysis, the effectiveness of the components of Light’s criteria, pleural cholesterol level, and pleural triglyceride level in differentiating exudates from transudates was evaluated. The most effective parameter for distinguishing exudates from transudates was found to be the protein ratio. Pleural cholesterol level was found to differentiate exudates from transudates with high efficacy, comparable to the protein ratio. Pleural cholesterol was observed to differentiate exudates from transudates with greater efficacy than pleural protein, pleural LDH, and the LDH ratio. The efficacy of pleural triglyceride level was found to be the lowest (Figure 3a,b, Table 2).

ROC curve analysis identified an optimal pleural cholesterol cut-off value of ≥50 mg/dL based on the maximum Youden Index. A cutoff value of ≥50 mg/dL for pleural cholesterol was highly effective in differentiating exudative effusion from transudative effusion (sensitivity 92.2%, specificity 94.9%, PPV 97.5% and NPV 84.8%). A cutoff value of ≥30 mg/dL for pleural triglyceride yielded a sensitivity of 60.0% and a specificity of 88.2%. Among the components of Light’s criteria, the protein ratio demonstrated the highest specificity, sensitivity, PPV, and NPV (Table 3).

To evaluate whether pleural fluid cholesterol remained independently associated with exudative pleural effusion after adjustment for other clinically relevant variables, a multivariable logistic regression analysis was performed (Table 4). Pleural fluid cholesterol ≥50 mg/dL remained a strong independent predictor of exudative pleural effusion (OR 126.03, 95% CI 34.38–462.06; *p* < 0.001). In addition, pleural LDH and the presence of malignancy were independently associated with exudative pleural effusion, whereas age and serum protein level were not significant predictors.

Light’s criteria showed a sensitivity of 99.2% in differentiating exudative effusions from transudative effusions; however, the corresponding specificity was lower at 84.4%. Overall, Light’s criteria misclassified 37 of the 235 transudative pleural effusions (15.6%) as exudates. In comparison, the use of a pleural cholesterol cut-off value of ≥50 mg/dL reduced the number of misclassified transudative pleural effusions to 12 (5.1%), whereas a cut-off value of ≥45 mg/dL resulted in 28 (11.9%) misclassified cases. These findings demonstrate that pleural cholesterol, particularly at a cut-off value of ≥50 mg/dL, substantially reduced the misclassification of transudative pleural effusions as exudates compared with Light’s criteria.

## 4. Discussion

This study demonstrated that pleural fluid cholesterol is a highly accurate biomarker for differentiating exudative from transudative pleural effusions. Although Light’s criteria maintained excellent sensitivity, pleural cholesterol provided substantially higher specificity and reduced the misclassification of transudative pleural effusions, particularly when a cut-off value of 50 mg/dL was applied. These findings support the potential clinical utility of pleural cholesterol as a complementary biomarker in the diagnostic evaluation of pleural effusions.

Costa et al. showed that in a total of 180 patients with pleural effusion, of whom 131 (72.7%) had exudative effusions according to etiology, a pleural cholesterol threshold of 45 mg/dL differentiated pleural exudates from transudates with an accuracy similar to that reported by Light et al. [7,11]. Pleural cholesterol levels of >45 mg/dL were shown to identify exudative effusions with 99% sensitivity and 98% specificity. These results, obtained without the need for simultaneous blood sampling, were reported to be usable as an alternative to Light’s criteria.

Similarly, Gautam et al., and more recently, Pg et al., Hamal et al., and Dhandapani et al. reported in studies involving small patient populations that when a cutoff value of 45 mg/dL was used for pleural cholesterol, exudative fluids could be distinguished from transudative fluids with high sensitivity and specificity [12,13,14,15]. They suggested that pleural cholesterol was more effective than Light’s criteria and recommended its routine use in the differentiation of exudates and transudates in patients with pleural effusion.

More recently, Majmundar et al., while studying 140 patients with pleural effusion, reported that a pleural fluid cholesterol cutoff value of 45 mg/dL yielded 97.95% sensitivity, 95.23% specificity, 97.95% PPV, and 95.23% NPV for exudate–transudate differentiation [16].

Rustogi et al., in a study of 101 patients with pleural effusion, found that mean pleural cholesterol levels were 27.8 ± 7.84 and 70.76 ± 22.35 mg/dL in transudative and exudative fluids, respectively [17]. With a cutoff value of 45 mg/dL for pleural cholesterol, they found 98% sensitivity and 95% specificity in differentiating exudates from transudates. In conclusion, they emphasized that pleural cholesterol performs as well as Light’s criteria in differentiating transudates from exudates.

Rufino et al. evaluated 100 patients and reported 79 cases to be definitively diagnosed as exudative effusion [17]. They reported a mean cholesterol level of 90.39 mg/dL in exudative fluid. A cutoff value of 50 mg/dL for exudative fluid yielded a sensitivity of 97.22%, specificity of 85.71%, PPV of 98.59%, and NPV of 75%. Their findings were in line with the mean cholesterol level in exudative fluid reported in the present study (91.1 mg/dL). In our study, specificity and PPV were higher.

In a systematic review by Wilcox et al., involving 37 studies, a cutoff value of >55 mg/dL for pleural fluid cholesterol was found to identify exudates most accurately [18].

Recent international recommendations continue to endorse Light’s criteria as the standard initial approach for differentiating transudative and exudative pleural effusions because of their excellent sensitivity [2]. However, updated pleural disease guidelines also recognize that pleural fluid cholesterol may be useful in specific clinical settings, particularly when serum samples are unavailable or additional biochemical evaluation is required [2]. Our findings further support the complementary role of pleural fluid cholesterol by demonstrating that it substantially reduced the misclassification of transudative pleural effusions while maintaining high diagnostic accuracy.

In studies evaluating the effectiveness of pleural cholesterol in distinguishing transudates from exudates, a pleural cholesterol cutoff value of 45 mg/dL has most often been proposed. In most of these studies, the number of patients evaluated was <100. The sample size of only a small proportion of studies exceeded 100 patients. In the present study, which included a large number of patients, pleural cholesterol with a cutoff value of 50 mg/dL was shown to differentiate exudative fluids from transudative fluids with high specificity and PPV. In our study, when the cutoff value for pleural cholesterol was set at 45 mg/dL, specificity was found to decrease. Although the sensitivity of Light’s criteria is already close to 100%, its main limitation remains its relatively low specificity. Therefore, within the present study population, a cut-off value of 50 mg/dL appeared to be more appropriate than 45 mg/dL because of its higher specificity and PPV. Another important clinical drawback of Light’s criteria is that they incorrectly classify a high proportion of transudates as exudates. In the present study, Light’s criteria classified 15.6% of etiologically transudative cases as exudates. When the cutoff value for pleural cholesterol was set at 45 mg/dL, this rate decreased to 11.9%, whereas when the cutoff value was increased to 50 mg/dL, the rate further decreased to 5.1%. These findings indicate that pleural cholesterol substantially reduces the misclassification of transudative pleural effusions as exudates compared with Light’s criteria, particularly when a cut-off value of 50 mg/dL is applied.

Pleural cholesterol levels remained consistently elevated across the major exudative etiologies evaluated in this study, including malignant pleural effusion, parapneumonic effusion, and tuberculous pleural effusion, with no significant differences among these subgroups. In contrast, substantially lower pleural cholesterol levels were observed in patients with congestive heart failure, supporting the diagnostic stability of pleural cholesterol across the most common causes of exudative pleural effusion.

Previous studies have suggested that combining pleural cholesterol with conventional biochemical markers, particularly pleural protein and LDH, may improve the differentiation of exudative and transudative pleural effusions. In the present study, we further evaluated this issue using a multivariable logistic regression model that included age, serum protein level, pleural LDH, pleural cholesterol, and the presence of malignancy. Pleural cholesterol remained a strong independent predictor of exudative pleural effusion after adjustment for these variables, supporting its independent diagnostic value. These findings indicate that pleural cholesterol retains independent diagnostic value after adjustment for other clinically relevant variables and may serve as a useful complementary biomarker in the evaluation of pleural effusions.

### Study Limitations

This study has several limitations. First, its retrospective single-center design may have introduced selection bias and limited the generalizability of the findings. In addition, as with other retrospective diagnostic accuracy studies, the present study may have been susceptible to verification bias because the reference standard was established using routine clinical investigations rather than a single uniform confirmatory test for all patients. Furthermore, because this study was conducted at a tertiary referral center, the distribution of pleural effusion etiologies may not fully represent that of the general population, introducing potential spectrum bias. Second, although a relatively large cohort was included, subgroup analyses according to specific etiologies of pleural effusion were not performed. Third, the potential effects of concomitant conditions and treatments, particularly diuretic therapy in patients with congestive heart failure, could not be evaluated in detail due to incomplete retrospective data. In addition, serial measurements of pleural cholesterol levels were not available, and only baseline biochemical parameters obtained at the time of diagnostic thoracentesis were analyzed. Finally, although pleural cholesterol demonstrated high diagnostic accuracy, the optimal pleural fluid cholesterol cut-off value was derived from the present study population and was not internally validated using bootstrap resampling or cross-validation. Therefore, external multicenter validation in independent prospective cohorts is warranted to confirm the optimal cut-off value and establish its routine clinical applicability.

## 5. Conclusions

This study demonstrated that transudate-exudate differentiation can be achieved with high potential using pleural cholesterol measurement alone, without the need for a blood sample from the patient. In particular, in cases that are clinically considered transudative but are classified as exudative according to Light’s criteria, inclusion of pleural cholesterol measurement might substantially prevent false exudate classification. Therefore, in patients with pleural effusion, we recommend routine measurement of pleural cholesterol in addition to Light’s criteria, and we propose a pleural cholesterol cutoff value of ≥50 mg/dL for differentiating exudative from transudative effusions.

## Figures and Tables

**Table 1:** Comparison between the clinical and laboratory features of transudative and exudative effusions.

Variables	Transudative Pleural Effusion (*n*: 235)	Exudative Pleural Effusion (*n*: 516)	*p*-Value
Age (year)	76.2 ± 11.6	65.2 ± 16.2	<0.001
Sex F. *n* (%)	97 (41.3%)	194 (37.6%)	0.337
S. Glucose (mg/dL)	120 (99–168)	108 (91–194)	0.026
S. Albumin (g/dL)	31.7 ± 6.1	34.4 ± 5.8	<0.001
S. Protein (g/dL)	64.4 ± 8.1	67.6 ± 8.1	<0.001
S. LDH (IU/L)	251 (210–297)	245 (195–320)	0.413
CRP (mg/L)	1.9 (0.9–6.1)	7.8 (2.3–12.0)	<0.001
Sedimentation	43 ± 29	61 ± 31	0.005
P. Glucose (mg/dL)	134 (114–179)	108 (79–134)	<0.001
P. Albumin (g/dL)	12.5 ± 4.2	25.4 ± 6.0	<0.001
P. Protein (g/dL)	22.0 ± 6.9	44.6 ± 9.2	<0.001
P. LDH (IU/L)	96 (76–120)	389 (213–750)	<0.001
P. Cholesterol (mg/dL)	28.9 ± 13.5	91.1 ± 30.7	<0.001
P. Triglycerides (mg/dL)	16 (12–22)	33 (24–47)	<0.001
P. Erythrocytes, *n*	700 (130–2050)	3000 (1000–15,200)	<0.001
P. Leukocytes, *n*	389 (208–693)	1633 (769–3541)	<0.001

F: Female, S: Serum, P: Pleural, CRP: C-reactive protein, LDH: Lactic dehydrogenase. Data are presented as numbers, percentages, mean ± SD and median (IQR).

**Table 2:** Receiver operating characteristic (ROC) analysis of pleural fluid biomarkers and the components of Light’s criteria for differentiating exudative from transudative pleural effusions.

	Area	St. Error	95% Confidence Interval	*p*-Value
Protein ratio	0.985	0.004	0.977–0.992	<0.001
LDH ratio	0.953	0.008	0.938–0.968	<0.001
LDH > 2/3	0.956	0.008	0.940–0.972	<0.001
P. Protein	0.967	0.006	0.954–0.979	<0.001
P. Cholesterol	0.976	0.006	0.965–0.987	<0.001
P. Triglycerides	0.828	0.018	0.793–0.863	<0.001

**Table 3:** Diagnostic accuracy of pleural fluid biomarkers and the components of Light’s criteria for differentiating exudative from transudative pleural effusions at the optimal cut-off values.

Cutoff Values	Sensitivity (95% CI)	Specificity (95% CI)	PPV (95% CI)	NPV (95% CI)
Protein ratio > 0.5	0.944 (0.928–0.961)	0.949 (0.917–0.974)	0.976 (0.962–0.989)	0.884 (0.843–0.924)
LDH ratio > 0.6	0.893 (0.866–0.919)	0.884 (0.843–0.924)	0.946 (0.925–0.966)	0.786 (0.733–0.838)
P. LDH > 2/3	0.860 (0.830–0.889)	0.911 (0.874–0.947)	0.956 (0.937–0.974)	0.745 (0.689–0.800)
P. Cholesterol ≥ 45 mg/dL	0.946 (0.926–0.966)	0.881 (0.839–0.922)	0.946 (0.925–0.966)	0.881 (0.839–0.922)
P. Cholesterol ≥ 50 mg/dL	0.922 (0.898–0.945)	0.949 (0.917–0.974)	0.975 (0.960–0.989)	0.848 (0.802–0.893)
P. Triglycerides ≥ 30 mg/dL	0.602 (0.559–0.644)	0.882 (0.840–0.923)	0.923 (0.898–0.947)	0.484 (0.420–0.547)
P. Protein ≥ 30 g/L	0.939 (0.918–0.959)	0.849 (0.803–0.894)	0.914 (0.888–0.939)	0.865 (0.821–0.908)
P. Protein ≥ 30 g/L or P. Cholesterol ≥ 50 mg/dL	0.964 (0.947–0.980)	0.816 (0.766–0.865)	0.924 (0.902–0.951)	0.908 (0.871–0.944)
LIGHT	0.992 (0.984–0.999)	0.844 (0.797–0.890)	0.933 (0.910–0.955)	0.978 (0.959–0.996)

95% CI: 95% confidence interval, PPV: Positive predictive value, NPV: Negative predictive value.

**Table 4:** Multivariable logistic regression analysis identifying independent predictors of exudative pleural effusion.

Variables	OR	95% Confidence Interval	*p*-Value
Age (Years)	0.961	0.922–1.002	0.061
Serum protein level (g/L)	0.982	0.919–1.048	0.578
P. LDH (IU/L)	1.019	1.012–1.025	<0.001
P. Cholesterol (≥50 mg/dL)	126.033	34.378–462.056	<0.001
Presence of malignancy	246.384	24.741–2453.658	<0.001

OR: Odds ratio.

## Data Availability

All data generated or analyzed during this study are included in this article. The data will be available upon reasonable request (contact persons: elvan.senturktopaloglu@erdogan.edu.tr).
